# Functions of Jasmonic Acid in Plant Regulation and Response to Abiotic Stress

**DOI:** 10.3390/ijms21041446

**Published:** 2020-02-20

**Authors:** Jia Wang, Li Song, Xue Gong, Jinfan Xu, Minhui Li

**Affiliations:** 1 Inner Mongolia Key Laboratory of Characteristic Geoherbs Resources Protection and Utilization, Baotou Medical College, Baotou 014060, China; wwwmokcom@163.com (J.W.); hhhtsongli@126.com (L.S.); gongxue_2017@yeah.net (X.G.); xjf0815@163.com (J.X.); 2 Pharmaceutical Laboratory, Inner Mongolia Institute of Traditional Chinese Medicine, Hohhot 010020, China; 3 Qiqihar Medical University, Qiqihar 161006, China

**Keywords:** endogenous and exogenous, transcription factors and genes, signal transduction, plant hormone, crosstalk

## Abstract

Jasmonic acid (JA) is an endogenous growth-regulating substance, initially identified as a stress-related hormone in higher plants. Similarly, the exogenous application of JA also has a regulatory effect on plants. Abiotic stress often causes large-scale plant damage. In this review, we focus on the JA signaling pathways in response to abiotic stresses, including cold, drought, salinity, heavy metals, and light. On the other hand, JA does not play an independent regulatory role, but works in a complex signal network with other phytohormone signaling pathways. In this review, we will discuss transcription factors and genes involved in the regulation of the JA signaling pathway in response to abiotic stress. In this process, the JAZ-MYC module plays a central role in the JA signaling pathway through integration of regulatory transcription factors and related genes. Simultaneously, JA has synergistic and antagonistic effects with abscisic acid (ABA), ethylene (ET), salicylic acid (SA), and other plant hormones in the process of resisting environmental stress.

## 1. Introduction

Plants often endure biotic stress (pathogens, herbivores, or parasitic microorganisms) and abiotic stress (cold, drought, salinity, light, or wounding) [1,2]. To survive and reproduce, plants not only need to grow and develop but also tolerate environmental stress. There are numerous benefits of maintaining a balance between plant growth and stress tolerance. Farmers associate abiotic stress with considerable economic losses. Phytohormones play key roles in plant growth and development. In addition, many signaling networks that regulate stress responses have been identified [3,4]. Plant hormones, which are natural and non-toxic compounds, could be applied as safe and environmentally friendly chemical control agents. Jasmonates (JAs) are derivatives of fatty acids, including key compounds such as jasmonic acid (JA), methyl jasmonate (MeJA), and jasmonate isoleucine conjugate (JA-Ile) [5]. The core of the JA chemical structure is 3-oxo-2-2′-*cis*-pentenyl-cyclopentane-1-acetic acid, an endogenous signaling molecule involved in diverse developmental processes that was originally considered a stress-related hormone in higher plants [6,7]. Over the last decade numerous genes and transcription factors (TFs) involved in the JA biosynthesis and the signal transduction pathway have been identified, including various inhibitors and activators involved in environmental signaling [8,9]. There are numerous good reviews on research on JA biosynthesis [5,10,11]. The present review avoids repeating an introduction of JA synthesis; instead, it focuses on JA functions in signaling pathways that mediate responses to abiotic stress. In addition, the authors review diverse phytohormones including abscisic acid (ABA), ethylene (ET), salicylic acid (SA), gibberellin (GA), auxin (indole-3-acetic acid, IAA), and brassinosteroids (BR), which participate in complex signaling networks with JAs in response to abiotic stress.

## 2. JA-Mediated Abiotic Stress Responses

**Figure 1:**
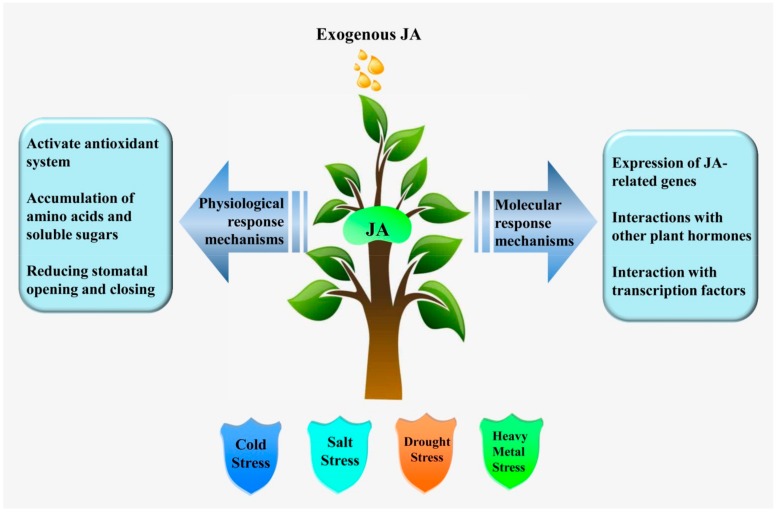
The role of jasmonic acid (JA) in plant response to abiotic stress.

Jasmonic acid is a plant-signaling molecule closely associated with plant resistance to abiotic stress. In abiotic stress, JA is usually involved in physiological and molecular responses. Physiological responses often include activation of the antioxidant system (superoxide anion radical, peroxidase, NADPH-oxidase) [12], accumulation of amino acids (isoleucine and methionine) and soluble sugars [13], and regulation of stomatal opening and closing [14]. Molecular responses often involve the expression of JA-associated genes (*JAZ, AOS1, AOC, LOX2,* and *COI1*) [15,16], interactions with other plant hormones (ABA, ET, SA, GA, IAA, and BR) [3,4], and interactions with TFs (MYC2 and bHLH148) [17,18]. Possible mechanisms of JA in abiotic stress tolerance are shown in Figure 1. In this section, we discuss the role of JA signaling in regulating plant responses under different environments.

### 2.1. Cold Stress

**Figure 2:**
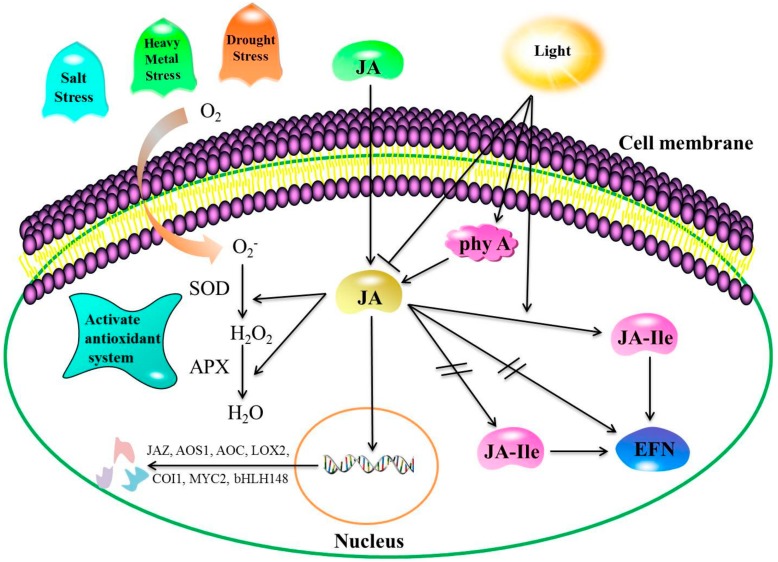
Response mechanism of endogenous JA to abiotic stress. Note: Positive regulatory actions or under light conditions are indicated by arrows and by lines and bars under dark conditions. Double slashes indicate that the process cannot proceed. Salt, drought, or heavy metal stress conditions induce oxidative stress due to elevated reactive oxygen species (ROS) generation levels. The JA produced facilitates stress tolerance by modulating major enzymatic components (SOD and APX) of antioxidant defense systems. In light, the secretion of extra-floral nectar (EFN) is promoted by JA and jasmonate isoleucine conjugate (JA-Ile). Conversely, no light inhibits the secretion of EFN by JA, but not JA-Ile. Far-red (FR) light induces phytochrome A (phyA) and activities of the JA singling pathway. SOD: superoxide dismutase; APX: ascorbate peroxidase.

Low temperature stress is a considerable limiting factor for plant growth and development, with a major influence on the geographical distribution of plants. There are two forms of low temperature stress: chilling and freezing stress, defined as plant injury caused by temperatures above and below 0 °C, respectively [19,20]. Plants have evolved complex tolerance mechanisms against such stress factors, including the expression of hormone-related genes and the accumulation of cold-induced stress-related proteins, amino acids, and soluble sugars to stabilize the subsequent cell membrane damage [21]. Low temperature conditions can induce the expression of JA biosynthesis genes, including allene oxide cyclase (*AOC*), allene oxide synthase1 (*AOS1*), and lipoxygenase2 (*LOX2*). Jasmonic acid positively regulates downstream cold-responsive genes, which are also upregulated by the C-repeat binding factor (CBF) transcriptional pathway to enhance cold tolerance [15]. Recent studies conducted on bananas have shown that two MYC2 TFs are activated rapidly following the exogenous application of MeJA in the course of cold storage. In addition, MeJA significantly enhances the expression of inducer of CBF expression (ICE-CBF) cold-responsive pathway genes [17]. Such findings demonstrate that the MaMYC2 transcription factor participates in MeJA-induced chilling tolerance in banana fruit in coordination with *MaICE1*. In addition, exogenous JA treatment can reduce lipoxygenase activity and increase antioxidant synthesis to enhance cold tolerance in plants. Furthermore, Li et al. [22] observed that the expression of *CBF*, late embryogenesis abundant (*LEA*), and dehydration-responsive element binding (*DREB1*) in *Zoysia japonica* increased under chilling stress, along with ABA and JA concentrations. Cao et al. [23] also found that superoxide dismutase (SOD), catalase (CAT), and ascorbate peroxidase (APX) activities in MeJA-treated loquat fruit increased in the course of loquat fruit storage, while lipoxygenase activity decreased (Figure 2).

### 2.2. Drought Stress

Climate change is leading to global warming and more frequent and/or extreme drought events in many important agricultural regions globally. The impact of drought stress on crops is one of the major reasons for reduction in crop yield reduction and even crop failure, reducing yields from many crops by more than 50% [24]. Overall, the effects of drought stress include suppressed plant growth [25,26], reduced photosynthetic rates [27], and accelerated leaf senescence [28,29]. In addition, drought stress can trigger oxidative reactions, induce membrane lipid accumulation, and induce antioxidant enzyme expression [30,31]. Jasmonic acid can minimize water loss by regulating stomatal opening and closing in *Arabidopsis thaliana* [32]. The concentrations of endogenous JAs increase rapidly following drought stress, and then return to the baseline levels if stress periods are prolonged. In addition, numerous genes and TFs associated with drought stress are expressed following drought stress. Jasmonate ZIM-domain proteins (JAZ) are regulators, typically repressors, in the JA signaling pathway. Fu et al. [33] demonstrated that *OsJAZ1* plays a negative regulatory role in rice drought stress tolerance, particularly in relation to the ABA and JA signaling pathways. In addition, Seo et al. [18] found that OsbHLH148, a basic helix–loop–helix protein, acts as a transcriptional regulator and up regulates *OsDREB1* and *OsJAZ,* which are involved in drought stress responses and the JA signaling pathway, respectively. Furthermore, Ge et al. [34] reported that in a drought-tolerant *Prunus armeniaca* genotype, transient JA accumulation could promote leaf senescence, prevent excessive water loss, and improve plant survival under soil drought conditions. Conversely, the exogenous application of JAs could alleviate drought stress associated damage in *P. armeniaca*. Foliar application of MeJA on soybean leaves can enhance water stress tolerance capacity, and further analysis showed increased levels of sugars, phenolic compounds, and flavonoids [35]. Such findings indicate that both endogenous and exogenous JAs participate in drought stress tolerance in plants.

### 2.3. Salt Stress

Salt stress interferes with plant metabolism, leading to oxidative stress, malnutrition, membrane disorders, and genotoxicity [36,37]. Both endogenous and exogenous JA can enhance plant salt stress tolerance [38]. Pedranzani et al. [39] analyzed changes in endogenous JAs under salt stress in tomato cultivars. According to their findings, the difference in lipid kinase activity between the two salt-tolerant cultivars studied was associated with salt stress tolerance, and not JA synthesis. Abouelsaad et al. [40] found that endogenous JA enhanced salt tolerance in tomato, mainly through homeostasis maintenance among reactive oxygen species (ROS). However, other studies have reported that exogenous JA treatments reduced salt-induced damage to various plants via increased photosynthetic rates, proline contents, ABA levels [41], and antioxidant enzyme activity [42], or via reductions in Na^+^ accumulation rates in shoots [43]. Shahzad et al. [44] observed that exogenous JA could improve Na^+^ exclusion in the root by decreasing Na^+^ uptake, facilitating surface salt stress tolerance in two maize genotypes. During the first phase of salt stress, JA levels increase, and could be involved indirectly in leaf growth inhibition in salt-sensitive genotypes. Qiu et al. [45] reported that three days of exposure to exogenous JA decreased the concentrations of malondialdehyde (MDA) and hydrogen peroxide (H_2_O_2_) in wheat seedlings significantly, improving the tolerance of wheat seedlings to salt stress. In addition, the transcript levels and SOD, peroxidase, CAT, and APX activities increased significantly. Such results indicate that JA could facilitate salt stress tolerance by enhancing the concentrations of antioxidative compounds and antioxidant enzyme activity (Figure 2).

### 2.4. Heavy Metal Stress

Heavy metals pollute the environment and impair plant growth and development [46]. Both fresh weight and photosynthetic pigment concentrations decreased in *Suaeda glauca* and *A. thaliana* exposed to high lead (Pb), nickel (Ni), cadmium (Cd), and manganese (Mn) concentrations. Many of these metals have no beneficial functions in plants, and may in fact be toxic to plants even at very low levels [47]. Zhao et al. [48] compared Cd stress responses in wild-type and JA-deficient mutant *spr2* tomatoes and observed that Cd concentrations in roots and leaves increased more at higher doses of CdCl_2_, particularly in *spr2* plants. The results demonstrated that a lack of endogenous JA could enhance the sensitivity of tomato seedlings to Cd. In addition, according to Sirhindi et al. [49], the exogenous application of JA before NiCl_2_ stress could enhance *Glycine max* seeding tolerance to Ni^2+^ stress. Furthermore, they revealed that JA protected the seedlings by regulating the antioxidant machinery and protecting DNA synthesis of total proteins, while Azeem [50] revealed that exogenous supplementation of JA alleviated the adverse effects of oxidative stress on growth, biomass production, and protein concentrations in Ni-treated plants by further increasing antioxidant enzyme activity. External JA supplementation could minimize CD accumulation rates in faba bean roots, shoots, and leaves not only by enhancing osmotic and antioxidant activity, but also by inhibiting H_2_O_2_, and MDA accumulation [51]. Noriega et al. [52] revealed that JA inhibited lipid peroxidase activity by activating ascorbate or glutathione antioxidant machinery. In addition, the significant increase in HO-1 antioxidant enzyme activity they observed under heavy metal stress could be regulated strictly by ROS homeostasis. Such findings indicate that JAs regulate plant responses to heavy metal stress by regulating their antioxidant systems (Figure 2).

### 2.5. Light Stress

Light is a key regulator of JA biosynthesis and signal transduction [53,54]. Radhika et al. [55] observed that both light and JA influenced extra-floral nectar (EFN) secretion: JA decreased EFN secretion in the dark, but induced EFN secretion under light conditions. Conversely, JA-Ile can enhance EFN secretion under light conditions, but does not decrease EFN secretion in the dark (Figure 2). In a JA-free *hebiba* mutant of *Oryza sativa*, JA or its precursor, 12-oxophytodienoic acid, could rescue the development of plants grown under both dark and red light conditions. Therefore, lack of JA potentially results in the phenotypic effects of the mutation [56]. Robson et al. [16] showed that *A. thaliana* mutants with reduced JA biosynthesis and signaling levels exhibited reduced levels of responses to high irradiance under far-red (FR) light. The results indicated that coronatine-insensitive1 (COI1), a central component of JA signaling, influenced FR light-induced expression of transcription factor genes, and that JA suppressed the expression. Notably, COI1-mediated degradation of JAZ1-glucuronidase (JAZ1-GUS) in response to JA treatment required phytochrome A (phyA). Overall, the findings indicate that the signaling activities of both phyA and JA function through the degradation of the JAZ1 protein, and that both are required in plant responses to light stress. Chen et al. [57] revealed that treatment with exogenous MeJA enhances the interaction between FR-insensitive 219 (FIN219) and the C terminus of cryptochrome 1 (CCT1) under blue light. Further investigations revealed that cryptochrome1 and FIN219 have a mutually antagonistic relationship, suggesting that the relationship between the JA signaling and blue light signaling pathways plays a key role in seedling development and stress responses. Liu et al. [58] found that JA pretreatment reduced the adverse effects of UV-B on photosystem II function significantly by increasing the effective photosystem II quantum yield, the capture efficiency of excitation energy in reaction center, and the photosynthetic electron transport rate, and by decreasing nonphotochemical quenching in wheat seedlings. The results confirm that exogenous JA could counteract the negative effects of UV-B stress on wheat seedlings. In *Nicotiana*, UV-B irradiation induces overlapping biochemical changes and the transcription of genes, including the JA biosynthesis and signaling pathways [59]. In addition, Demkura et al. [60] concluded that UV-B: (1) induces phenolic compound production via both JA-dependent and JA-independent pathways, and (2) enhances sensitivity to JAs, leading to enhanced expression of the wound-response gene, trypsin proteinase inhibitor.

### 2.6. Other Stress Factors

Ozone (O_3_), a major photochemical oxidant, causes severe damage to plants. After ozone treatment, endogenous JA concentrations increase significantly in wild-type *A. thaliana*. However, exogenous MeJA can inhibit O_3_-induced programmed cell death. A novel O_3_-sensitive and JA-insensitive *A. thaliana* mutant, O_3_-sensitive and jasmonate-insensitive 1 (*oji1*), has been isolated previously. JA is typically a protective compound under stress conditions. Higher O_3_ concentrations induce high ET emission levels in *oji1* [61,62]. Grantz et al. [63] reported that MeJA acts as an antiozonant in Pima cotton (*Gossypium barbadense*) and interacts synergistically with ET at very high O_3_ concentrations (685 ppb). Conversely, this is accompanied by antagonism to ET. In addition, Kaya et al. [64] found that *Nicotiana tabacum* treated with 45 μM JA had greater MDA and pigment (chlorophyll and carotenoids) concentrations, antioxidant activity (catalase, ascorbate peroxidase, glutathione S-transferase and glutathione reductase), and phytohormone (ABA and IAA) levels, but less herbicide residue (imazapic). Consequently, they proposed that exogenous JA regulates stress responses in tobacco plants exposed to herbicides. The plant circadian clock enables plants to measure day length and adapt to changes in circadian rhythm. Furthermore, circadian stress influences ROS- and JA-associated gene expression. Nitschke et al. [65] proposed that circadian stress activated the JA pathway in cytokinin-deficient *A. thaliana* plants. Notably, the induction of *MYC2* and *JAZ1* has also been detected in clock mutants *cca1-1, lhy-11*, and *elf3-9*, indicating that strong circadian stress responses are commonly associated with activated JA pathways.

In summary, both endogenous and exogenous JA participate in plant responses to abiotic stress. The JA regulatory mechanisms in response to cold, drought, salinity, heavy metal, light, O_3_, imazapic, and circadian stress are listed in Table 1.

## 3. Interactions between JA and Plant Hormone Pathways under Abiotic Stresses

**Figure 3:**
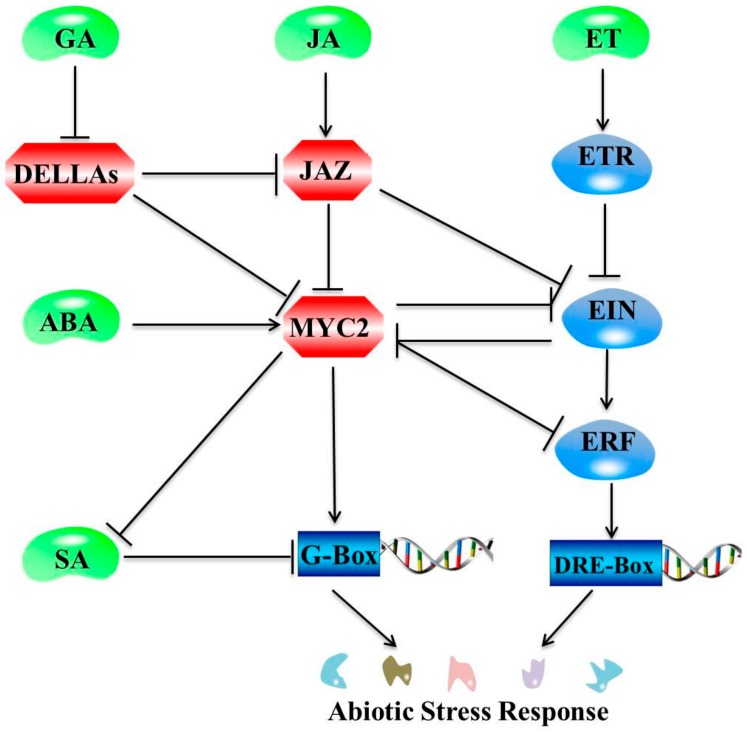
Schematic diagram representing crosstalk of JA with other plant hormone signaling pathways. Note: Positive and negative regulatory actions are indicated by arrows and lines with bars, respectively. MYC2 is the major component involved in interactions between JA and gibberellin (GA). DELLAs interact with JAZ repressors, relieving MYC2 from JAZ repression, and facilitate JA-mediated defense responses by the activation of MYC2. MYC2 is also positively regulated by ABA. Conversely, MYC2 inhibits salicylic acid (SA) regulation of abiotic stress response genes. The JAZ inhibition of EIN mediates JA and ET signaling synergy in plant resistance, whereas the reciprocal counteraction between MYC2 and EIN mediates JA and ethylene (ET) signaling antagonism.

Interactions among plant hormone signals are at the core of plant responses to biotic and abiotic stress factors. JA does not have an independent regulatory role but works within a complex signal network with other phytohormone signaling pathways, such as those of ABA [66,67], ET [68], SA [69], GA [70], IAA [71], and BR [72]. Consensus has been reached across numerous studies on plant responses to biotic and abiotic stress factors based on (1) the convergence of signaling pathways involved in transcription of multiple stimulus responses that are under the control of overlapping genes; and (2) the hormonal pathways that interact through stress responses to regulate different environmental stresses [73]. In response to dehydration stress, diverse phytohormones (ABA, JA, SA, GA, IAA, and ET) acted jointly to upregulate 859 genes identified using whole-genome transcriptome analyses in *A. thaliana* significantly [74]. An overview of crosstalk between JA and other major plant hormones for abiotic stress tolerance is presented in Figure 3. The following discussion explores recent studies on the mechanisms by which JAs and other plant hormones respond to abiotic stress.

### 3.1. Interactions between JA and ABA Pathways in Response to Abiotic Stresses

As a major phytohormone regulating abiotic stress responses, ABA interacts with the JA signaling pathway to induce plant physiological responses to mitigate the effects of various abiotic stress factors [75]. The findings of several studies suggest that JA and ABA signal transduction pathways exhibit both synergistic and antagonistic regulation characteristics [76,77]. JA interacts with ABA to tolerate chilling stress [78]. Yoshikawa et al. [79] demonstrated that JAs, ABA, and polyamines could be associated with low temperature stress responses in apple. Many key genes, such as *CBF, LEA*, and *DREB*, have been shown to respond to low temperature stress in the ABA and JA signal transduction pathways. Li et al. [22] reported that low temperature increased JA and ABA concentrations, while also inducing the upregulation of *ZjCBF*, *ZjLEA*, and *ZjDREB1* in *Z. japonica* leaves.

The bHLH transcription factor, MYC2, a major regulator of the core JA signaling mechanism, participates in the ABA signaling pathway in response to drought stress [80,81]. The same transcription factor also regulates *AOC1*, a key JA biosynthesis gene, at signal intersections of the ABA/JA signaling pathways [82,83]. OsbHLH148 transcript levels increased rapidly following treatment with MeJA or ABA, and abiotic stress factors including drought, salinity, and cold stress. Expression profiling analyses of transgenic versus wild-type rice have identified *OsDREB* and *OsJAZ* genes that are upregulated by OsbHLH148 over-expression [18]. Proline accumulation under water stress appears to be an ABA-independent response, as both JA-deficient (*jar1-1*) and JA-insensitive (*jai1*) *A. thaliana* lines accumulate similar levels [84]. This indicates that the JA and ABA signaling pathways coordinate to regulate each other’s responses to drought, salinity, cold, and water stress. Octadecanoid-responsive AP2/ERF-domain transcription factor 47 (*ORA47*) acts as a gene target in ABA and JA biosynthesis when *A. thaliana* is subjected to water stress [85].

Many studies have reported that ABA and JA are involved in salt stress responses in plants. Treatment with JA further increased ABA concentrations in rice under salt stress. In addition, endogenous GA concentrations increased following treatment with JA [86]. Furthermore, according to Kim et al. [87], applying JA after NaCl treatment, rather than before, could increase endogenous ABA concentrations. Wang et al. [88] reported that during plant growth, JA and ABA concentrations generally increased under salt stress, while SA and IAA concentrations declined. Brossa et al. [89] proposed that the interaction of JA and ABA signaling during the regulation of antioxidant status is responsible for acclimating plants to osmotic stress.

### 3.2. Interactions between JA and Ethylene Pathways under Abiotic Stresses

JAs and ET play regulate plant defense against cold, drought [90], and salinity stress [91] through coordination and antagonism. Ethylene response factors (ERFs) conferring abiotic stress tolerance are induced not only by ethylene, but also by the JA signaling pathway. Therefore, they could facilitate crosstalk among abiotic stress response pathways [67]. The relationship between AP2/ERF TFs and the JA signaling pathway was first identified in *Catharanthus roseus* [92]. Lorenzo et al. [93] reported that ERF1 acting downstream of the ET and JA pathways could be central to the regulation of plant defensive responses. Pre et al. [94] found that the AP2/ERF-domain transcription factor ORA59 in *A. thaliana* was an essential integrator of the JA and ET signal transduction pathways, providing novel insights into the molecular mechanisms underlying in the crosstalk between JA and ET. In soybean, *GmERF7* expression decreased under cold treatment, although the expression of the gene was induced by drought, salt, or treatment with MeJA, ET, or ABA. [90]. In *C. roseus*, the octadecanoid-derivative responsive catharanthus AP2-domain protein (ORCA), inducible by JAs, belongs to the AP2/ERF-domain family and links plant salinity stress responses to changes in metabolism [91].

In *A. thaliana*, O_3_-induced cell death is regulated by both the JA and ET signal transduction pathways. Grantz et al. [63] reported that at very high O_3_ concentrations, synergistic O_3_ and MeJA interaction took place in Pima cotton. However, the plant responses to O_3_ were not mediated by the ET and MeJA signaling pathways. Stress caused by excess or inadequate nutrients is also regulated by both the JA and the ET signaling pathways. Although selenium (Se) is a vital plant nutrient, it can be toxic when present in excess concentrations. Recent studies have found that JA and ET synergistically regulate Se-induced stress responses [95]. JA and ET, therefore, act synergistically in plant responses to abiotic stress.

### 3.3. The Interactions between JA and SA Pathways under Abiotic Stresses

As an endogenous growth regulator, SA regulates plant physiological processes. It also plays an important role in the plant response to adverse environmental conditions such as cold [96,97,98], drought [99], salinity [43] and light [100]. Górnik et al. [96] reported that treatment of seeds with SA or JA could improve the resistance of seedlings to chilling, while Ilyas et al. [97] demonstrated that exogenous application of SA and JA could enhance drought stress tolerance in wheat, although JA was more effective than SA. However, the combined application of SA and JA did not significantly influence plant growth. Sayyari et al. [98] found that hydrophilic total antioxidant activity in pomegranates fruits increased following treatment with both MeJA and methyl salicylate (MeSa), while there were no significant changes in lipophilic total antioxidant activity. The findings suggest that both MeJa and MeSa have potential postharvest applications in reducing chilling injury. The mechanism by which JA and SA protect against salt stress is via the induction of protein-coding gene expression [43]. In addition, according to Farhangi-Abriz et al. [101] exogenous SA and JA decreased the concentration of Na^+^ in soybean under different salt stress levels, although there was no significant effect on the concentrations of Na^+^ in the absence of salt stress. Therefore, JA has a greater effect on Na^+^ reduction than SA. The SA and JA signaling pathways share a key regulator, glutaredoxin GRX480, which mediates redox regulation of proteins because of their capacity to catalyze disulfide transitions [102]. Research on *Nicotiana attenuata* found that mitogen-activated protein kinase 4 (MAPK4) is a negative regulator in the SA signaling pathway but is a positive regulator in the JA signaling pathway in response to light stress [100]. Coordinated regulation between JA and SA signaling pathways, therefore, involves multiple factors, including GRX480 or MAPK4. In addition, exogenous application of SA and JA can enhance abiotic stress responses.

### 3.4. Interactions between JA and other Plant Hormone Pathways under Abiotic Stress

Numerous studies have demonstrated that plant growth and development are regulated in a coordinated manner by the JA and IAA signal transduction pathways. However, no study has explored coordinated abiotic stress responses involving JA and IAA. Similarly, plant growth and defense are regulated through synergistic and antagonistic interactions between the JA and GA pathways. However, plant stress responses are often at the expense of growth inhibition [103]. In the presence of JA, JAZs are degraded to release DELLAs, plant growth repressors whose degradation is promoted by GA and which confer plants with elevated resistance to necrotrophs via potentiating JA signaling [104]. Similarly, the JA and ABA signaling pathways play an antagonistic role in plant growth and development, while operating synergistically in response to environmental stress. During plant responses to salt stress, ABA and JA typically increase, whereas IAA and SA decline. The Gretchen Hagen 3 (*ZmGH3*) gene in *Zea mays* is responsive to several abiotic stress factors (salinity, drought, and Cd) and major stress-related hormones (ABA, SA, and JA) [88]. The MYC2 TF of JAZs are key intermediates in coordinated regulation activities taking place via the JA and ABA signaling pathways, and they influence plant growth and defense activities [73]. JA inhibits overall plant growth, while BR promotes above ground growth. Coordination between the JA and BR signal transduction pathways is also involved in the balance between plant growth and defense. The crosstalk of JA with other plant hormones under environmental stress conditions is achieved primarily by regulating plant growth.

## 4. Conclusions

The mechanisms of action of JA are different under different environmental stress factors due to the diversity of plant hormone signals and interactions among different signals. Numerous genes (*JAZ, AOS1, AOC, LOX2,* and *COI1*) and TFs (MYC2 and bHLH148) are involved in the core JA signaling pathway as activators or repressors, mediating responses to environmental stress signals. As illustrated in Figure 3, the JAZ-MYC module plays a central role in the JA signaling pathway through the integration of regulatory TFs and associated genes. However, research on plant perception of different abiotic stress signals and the initiation of the JA response has not been systematic.

JAs function synergistically and antagonistically with ABA, ET, SA, and other plant hormones to tolerate environmental stress. The process is often accompanied by the regulation of plant growth and development hormones [103,105]. Further systematic research on the mechanisms of interaction of JA signaling with other plant hormones in response to abiotic stress is required.

In recent years, advancements in genomics, transcriptomics, and proteomics have provided more clues on such complex gene and protein interaction networks. Such tools also facilitate the study of the regulatory mechanisms of plant hormones exclusively, and of crosstalk under different environmental stress factors. However, plant hormone signaling networks are complex and unpredictable, particularly under complex and volatile environments. Because of the large quantity of data and the complexity of plant hormone systems, JA regulation under environmental stress is yet to be clarified. Understanding the signal transduction mechanism of JA under different environmental stress factors could facilitate the improvement of plant tolerance to environmental stress in the future, with potential improvements in plant survival and crop yield.

## Figures and Tables

**Table 1:** Regulation mechanism of endogenous and exogenous JAs in response to abiotic stress in plants.

Type of Stress	Plant Species	JA	Regulation Mechanism	Reference
Freezing	*Arabidopsis thaliana*	Endogenous	Positively regulated the C-repeat binding factor (CBF) transcriptional pathway to up-regulate downstream cold-responsive genes	[15]
Chilling	*Musa acuminata*	Endogenous	Induced MaMYC2 and inducer of CBF expression (ICE-CBF) cold-responsive pathway gene expression, including *MaCBF1*, *MaCBF2*, *MaCOR1*, *MaKIN2*, *MaRD2,* and *MaRD5*	[17]
Chilling and freezing	*Zoysia japonica*	Endogenous	Up-regulated *ZjCBF*, *ZjDREB1,* and *ZjLEA* expression	[22]
Chilling	*Eriobotrya japonica*	Exogenous (10 μM)	Enhanced antioxidant enzyme activity and higher unsaturated/saturated fatty acid ratio	[23]
Drought	*Arabidopsis thaliana*	Endogenous	Produced higher 12-OPDA levels and reduced stomatal aperture	[32]
Drought	*Oryza sativa.*	Endogenous	OsJAZ1 was a negative regulator via the abscisic acid (ABA)-dependent and JA-dependent pathways.	[33]
Drought	*Oryza sativa.*	Endogenous	OsbHLH148 acted on the JA signaling pathway with *OsJAZ1* and *OsCOI1*, constituting an OsbHLH148–OsJAZ–OsCOI1 signaling module	[18]
Drought	*Prunus armeniaca*	Exogenous (50 µM)	Increased malondialdehyde (MDA) levels and promoted leaf senescence	[34]
Drought	*Glycine max*	Exogenous (20 μM)	Increased cell wall fractionation, saturated and unsaturated fatty acid, flavonoid, phenolic acid, and sugar fraction content	[35]
Salt	*Lycopersicon esculentum*	Endogenous	Increased lipoxygenase (LOX), AOS-mRNA, and Pin2-mRNA accumulation	[39]
Salt	*Solanum lycopersicum*	Endogenous	Activated both enzymatic and non-enzymatic ROS antioxidants	[40]
Salt	*Zea mays*	Exogenous (25 μM)	Improved Na^+^ exclusion by decreasing Na^+^ uptake	[44]
Salt	*Triticum aestivum*	Exogenous (2 mM)	Decreased the concentration of MDA and H_2_O_2_, and increased the transcript levels and activities of SOD, POD, catalase (CAT), and APX	[45]
Heavy metal (cadmium)	*Lycopersicon esculentum*	Endogenous	JA played a positive regulatory role in tomato plant response to Cd stress by regulating the antioxidant system	[48]
Heavy metal (nickel)	*Glycine max*	Exogenous (1 μM and 1 nM)	Managed the antioxidant machinery and protected the DNA synthesis of total proteins to mitigate Ni stress	[49]
Heavy metal (nickel)	*Zea mays*	Exogenous (10 μM)	JA alleviated the negative impact of Ni-treated plants by improving the activity of antioxidant enzymes SOD, CAT, APX, GPX, and GR	[50]
Heavy metal (cadmium)	*Vicia faba*	Exogenous (10 μM)	Inhibited the accumulation of Cd, H2O2, and MDA, and enhanced osmolyte and antioxidant activities that reduce oxidative stress	[59]
Heavy metal (cadmium)	*Glycine max*	Exogenous (20 μM)	Augmented the activities of antioxidant enzymes CAT and SOD to Cd treatment	[51]
Light and darkness	*Phaseolus lunatus*	Endogenous	JA-Ile enhanced EFN secretion under light conditions, yet did not reduce EFN secretion in the dark	[55]
Light and darkness	*Oryza sativa*	Endogenous	JA and phytochrome A signaling were integrated through degradation of the JAZ1 protein	[56]
Far-red	*Arabidopsis thaliana*	Exogenous (50 μM)	Interaction of the photoreceptor CRY1 and the JA-conjugating enzyme FR-insensitive219/JAR1	[57]
UV-B	*Triticum aestivum*	Exogenous (1 and 2.5 mM)	Increased reaction centers’ excitation energy capture efficiency, effective PSII, and electron transport rate (ETR), and decreased NPQ	[58]
Ozone stress	*Arabidopsis thaliana*	Exogenous (100 μM)	Inhibited the spread of programmed cell death	[62]
Imazapic stress	*Nicotiana tabacum*	Exogenous (45 μM)	Increased antioxidant activity and phytohormone level and decreased MDA content	[64]
Circadian stress	*Arabidopsis thaliana*	Endogenous	Reduced the cell death phenotype	[65]

## Abbreviations

JA	Jasmonic acid
JAZ	Jasmonate ZIM-domain proteins
MeJA	Methyl jasmonate
JA-Ile	Jasmonate isoleucine conjugate
JAs	Jasmonates
TF	Transcription factor
ABA	Abscisic acid
ET	Ethylene
SA	Salicylic acid
GA	Gibberellin
IAA	Indole-3-acetic acid
BR	Brassinosteroids
NADPH	Nicotinamide adenine dinucleotide phosphate
bHLH148	basic helix-loop-helix protein 148
AOC	Allene oxide cyclase
AOS1	Allene oxide synthase1
LOX2	Lipoxygenase2
CBF	C-repeat binding factor
ICE-CBF	Inducer of CBF expression
SOD	Superoxide dismutase
CAT	Catalase
APX	Ascorbate peroxidase
JAZ	Jasmonate ZIM-domain proteins
ROS	Reactive oxygen species
MDA	Malondialdehyde
EFN	Extra-floral nectar
FR	Far-red
FIN219	Far-red insensitive 219
CCT1	C terminus of cryptochrome 1
ETR	Electron transport rate
Pb	Lead
Ni	Nickel
Cd	Cadmium
Mn	Manganese
Se	Selenium
HO-1	Hemeoxygenase-1
O_3_	Ozone
PIF3	Phytochrome-interacting factor 3
12-OPDA	12-oxo-phytodienoic acid
POD	Peroxidase
GPX	Glutathione peroxidase
GR	Glutathione reductase
CRY1	Cryptochrome 1
PSII	Photosystem II
NPQ	Non-photochemical quenching
EIN	Ethylene insensitive
AP2/ERF	APETALA2/Ethylene responsive factor
